# Mixotrophic Microalgae Biofilm: A Novel Algae Cultivation Strategy for Improved Productivity and Cost-efficiency of Biofuel Feedstock Production

**DOI:** 10.1038/s41598-018-31016-1

**Published:** 2018-08-21

**Authors:** Javad Roostaei, Yongli Zhang, Kishore Gopalakrishnan, Alexander J. Ochocki

**Affiliations:** 1 Civil and Environmental Engineering, Wayne State University, 5050 Anthony Wayne Dr., Detroit, MI 48202 USA; 2 Biological Sciences, Wayne State University, 5047 Gullen Mall, Detroit, MI 48202 USA; 3 Biochemistry & Molecular Biology, Wayne State University, 4263 Scott Hall, Detroit, MI 48202 USA

## Abstract

In this work, we studied a novel algae cultivation strategy, mixotrophic microalgae biofilm, to improve the productivity and cost-efficiency of algal biofuel production. In contrast to previous methods, this improved approach can achieve high productivity at low cost by harnessing the benefits of mixotrophic growth’s high efficiency, i.e., capable of subsisting on inorganic and organic carbons thus unaffected by limited light, and microalgae biofilm’s low harvesting cost. Our results, as one of the first studies of this type, proved that microalgae biofilms under mixotrophic condition exhibited significantly higher productivity and quality of biofuel feedstock: 2–3 times higher of biomass yield, 2–10 times higher of lipid accumulation, and 40–60% lower of ash content when compared to microalgae biofilms under autotrophic condition. In addition, we investigated the impact of cell-surface properties (hydrophobicity and roughness) on the growth activities of microalgae biofilms and found that the productivity of mixotrophic biofilms was significantly correlated with the surface hydrophobicity. Finally, our work demonstrated the applicability of integrating this novel cultivation method with wastewater for maximum efficiency. This study opens a new possibility to solve the long-lasting challenges of algal biofuel feedstock production, i.e., low productivity and high cost of algal cultivation.

## Introduction

Microalgae are among the most promising resources to provide multiple energy and environmental benefits such as bioenergy production, nutrients recovery and carbon sequestration^1–4^, yet the low productivity and high cost of algal cultivation impede advancement in their intensive applications^2,5^. The state-of-the-art algae cultivation approaches have primarily focused on algal cultures in open ponds. Although these systems are easily built, they are susceptible to light limitations and stresses that hamper algal growth beyond a cell concentration of 0.5 g/L^6–10^. Moreover, biomass harvesting and concentration are extremely costly due to low algal cell density^6–10^. Other studies have investigated closed photobioreactors, which have higher productivity. However, they are prohibitively expensive for large-scale applications due to high construction and operation/maintenance costs^6,11^. An alternative approach is immobilized cultivation, such as microalgae biofilm. Microalgae biofilm has three advantages: (1) resistance to growth stresses, (2) high cell density, and (3) low harvesting and concentration costs^12–18^. Another benefit of microalgae biofilm is the multiple-layer design of cultivation systems. Algae biofilm systems can be designed with multiple layers (horizontal, vertical, and rotating design), increasing the productivity per land area and the efficiency of land use^19–21^. Progress has been made in using microalgae biofilm for wastewater treatment in the form of various biofilm reactors^15,21–38^. However, these studies primarily focused on autotrophic conditions and are, thus, similar to open ponds, susceptible to light limitations resulting in low productivity.

Indeed, many microalgae species can grow under autotrophic, mixotrophic and heterotrophic schemes^39–42^. Among these three growth mechanisms, mixotrophic cultivation is particularly appealing because algae can grow under both autotrophic and heterotrophic metabolisms by using sunlight and inorganic/organic carbons^43^. Mixotrophic growth can maximize the usage of resources and eliminate problems associated with light limitations, achieving a higher growth rate and greater lipid content^39–42,44–47^. A number of studies have demonstrated the feasibility of growing microalgae planktonically under mixotrophic conditions for higher biomass and lipid yield. However, there still remain two challenges for such cultivation: (1) high harvesting and concentration costs and, (2) the susceptibility to stresses^39–42,44,45^. Noticeably, studies investigating microalgae biofilm under mixotrophic condition are very limited.

The overall goal of this research is to study a novel cultivation strategy, mixotrophic microalgae biofilm, for cost-efficient algal feedstock production. We hypothesize that this approach can reach high productivity at low-cost and is a more robust and cost-efficient system, as compared to current algae cultivation strategies. The underlying theory is that mixotrophic microalgae biofilm can harness the benefit of mixotrophic growth’s high efficiency, i.e., capable of subsisting on inorganic and organic carbons thus unaffected by limited light^6,48,49^. Additionally, microalgae biofilm has a high resistance to environmental stresses and, most importantly, the harvesting cost is low^13–15^. To test this hypothesis and evaluate the potential of mixotrophic microalgae biofilm in energy and environmental applications, three research objectives are included in this study: (1) characterizing the productivity and quality of algal feedstock under autotrophic and mixotrophic biofilm conditions; (2) defining cell-surface structures contributing to the formation and growth of microalgae biofilm; and (3) understanding the applicability of mixotrophic microalgae biofilm in wastewater-based algae cultivation.

## Results and Discussion

### Algal Biomass Productivity

Modified Bold 3N medium (MB3N) was used to exam the formation and growth activities of microalgae biofilms under different conditions including two microalgae species (*Chlorella vulgaris* and *Scenedesmus dimorphus*), two cultivation modes (autotrophic and mixotrophic), four supporting materials with different surface hydrophobicity (static water contact angle, 48 *ϴ*–74 *ϴ*), and three degrees of surface roughness (treated with 60-, 220- and 400-grit sandpaper). *Chlorella vulgaris* and *Scenedesmus dimorphus* are fresh water algae which are able to grow in wastewater^50,51^. The mixotrophic cultivation is based on the availability of organic carbon as an additional source for growth. In this study, glucose was used as the standard organic carbon source for mixotrophic cultivation.

**Figure 1:**
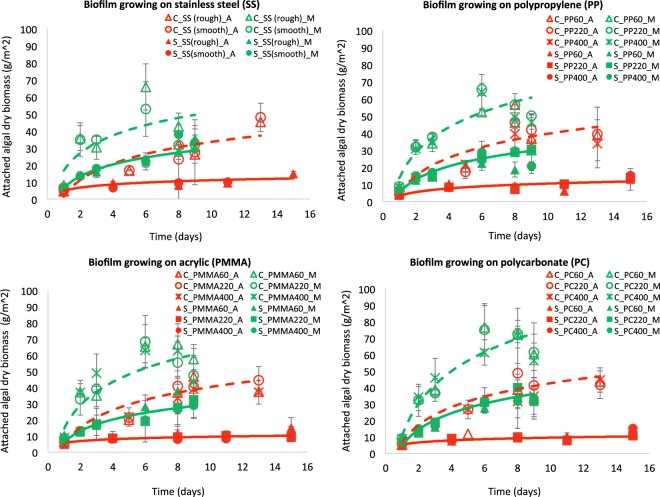
Dry algal biomass in microalgae biofilms under different cultivation conditions. Mixotrophic growth significantly (p < 0.001) promoted the formation and growth of microalgae biofilm. C, *Chlorella vulgaris*. S, *Scenedesmus dimorphus*. A, autotrophic cultivation. M, mixotrophic cultivation. SS, stainless steel. PP, polypropylene. PMMA, acrylic. PC, polycarbonate. 60, 220, 400, treated with 60-, 220-, and 400-grit sandpaper. For stainless steel, there were only two treatments: rough (treat with 220-grit sandpaper) and smooth (no treatment). Green and red solid line, the growth trend of *Scenedesmus dimorphus* under mixotrophic and autotrophic condition respectively. Green and red dash lines, the growth trend of *Chlorella vulgaris* under mixotrophic and autotrophic condition respectively.

As shown in Fig. 1, the results clearly show that mixotrophic biofilms, compared to autotrophic ones, can significantly promote biomass productivity (p < 0.001). The overall accumulation of algal biomass in mixotrophic biofilms was 2- and 3-time higher than that in autotrophic biofilms for *Chlorella vulgaris* and *Scenedesmus dimorphus* respectively, up to 75.2 g/m^2^ (*Chlorella vulgaris*) and 34.1 g/m^2^ (*Scenedesmus dimorphus*) under mixotrophic condition in 9 days while only 44.8 g/m^2^ (*Chlorella vulgaris*) and 12.3 g/m^2^ (*Scenedesmus dimorphus*) under autotrophic conditions in 13–15 days. In addition, the formation and growth of mixotrophic biofilms were more rapid, achieving the stationary phase in 3–5 days as compared to 6–8 days for autotrophic biofilms. The average daily productivity by the time reaching to the middle of the stationary stage was 12.2 g/m^2^-day (*Chlorella vulgaris*) and 5.3 g/m^2^-day (*Scenedesmus dimorphus*) for mixotrophic growth, while only 5.1 g/m^2^-day (*Chlorella vulgaris*) and 2.8 g/m^2^-day (*Scenedesmus dimorphus*) for autotrophic growth. These features are extremely attractive for real-world applications because they allows for a higher growth rate in a shorter cultivation timeframe which can improve the overall cost-efficiency.

The average productivities of algae cultivation have been reported ranging from 4–20 g/m^2^-day with a few reports greater than 30 g/m^2^-day for open ponds^52^ and 10–48 g/m^2^-day for closed photobioreactors^53^. Microalgae biofilms usually have lower biomass productivities (typically 1–5 g/m^2^-day) although higher productivity was reported^54–56^. This is probably due to the over-shading and nutrients limitation (particularly CO_2_ partitioning) to the underlying cells of algae biofilms since current studies of biofilm reactors have been primarily focused on autotrophic cultivation. In comparison, our results show that biomass yields of mixotrophic microalgae biofilms are very promising with average productivities of 5.3–12.2 g/m^2^-day, more than two times of commonly reported yields for microalgae biofilms and quite comparable to the average productivity in open ponds. One explanation could be that mixotrophic biofilms can maximize the utilization of all invested resources (sunlight and inorganic/organic carbons), thus reducing the effect of over-shading and nutrients limitation for higher yields. The other reason could be that mixotrophic condition offers an excess of carbon flux, promoting extracellular polysaccharide accumulation/release which can be catalyzed by C/N ratio^57,58^. The extracellular polysaccharide can provide many diverse benefits to biofilms’ formation and growth, including adhesion, protection and structural integrity^59^.

### Effects of Cell-Surface Properties on the Formation and Growth of Microalgae Biofilm

**Figure 2:**
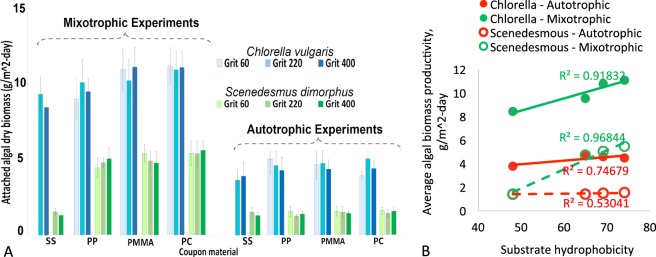
(**A**) The average daily biomass yields of algal biofilms growing on different substrates with different hydrophobicity and roughness. (**B**) The correlation between the average daily biomass yield and surface hydrophobicity. The formation and growth of mixotrophic microalgae biofilm was significantly correlated with the hydrophobicity of the supporting material.

The hydrophobicity (static water contact angle, *ϴ*) of stainless steel (SS), polypropylene (PP), acrylic (PMMA) and polycarbonate (PC) were measured as 48.0, 64.9, 69.0, and 74.0 respectively (SI, Fig. S1) with higher *ϴ* being more hydrophobic^60^. The average daily biomass yield tended to increase along with higher hydrophobicity for mixotrophic biofilms (Fig. 2A). Statistical analysis showed that the average biomass yield of mixotrophic biofilm was significantly correlated to the surface hydrophobicity for both *Chlorella vulgaris* (p = 0.041) and *Scenedesmus dimorphus* (p = 0.016) (Fig. 2B), with the growth efficiency being PC > PMMA > PP > SS. However, for autotrophic biofilms, the correlation was not statistically significant (p > 0.05). For the effect of surface roughness, no significant correlation between biofilms’ growth and surface roughness was observed (p > 0.05).

Biofilms are formed by the attachment of microorganisms on submerged surfaces in the aquatic environment^61^. Biofilm’s formation and growth depend on cell-surface properties, as particularly pertaining to surface hydrophobicity and surface roughness/pattern^62–66^. Due to limited studies, the impact of cell-surface structures on microalgae biofilm is unclear and inconclusive. Especially for mixotrophic microalgae biofilm, no information is available. For the effect of surface hydrophobicity, some researchers found positive correlation between surface hydrophobicity and biofilm formation and growth. They concluded that hydrophobic substrates could promote microalgae biofilms’ formation and growth^67–69^. The possible reason behind this is that algae cells are generally hydrophobic molecules which prefer to adhere to hydrophobic surfaces to minimize their contact with water^70^. Other studies, however, reported no or weak correlation^71,72^. The difference between these findings is probably attributed to the length of the growth period. Reports with positive correlation were usually observed for the initial colonization of algal cells to substrates, which is mainly determined by cell-surface properties. Findings with no/weak correlation, on the other hand, were obtained for relatively long-period cultivation where algal cells grow on top of each other and biomass accumulation is a function of other growth parameters such as nutrients concentrations, light availability, shearing effect, etc.^71,72^. As the growth period increases, the impacts of these growth parameters could eventually diminish the effect of the initial colonization. Our results found that, for autotrophic microalgae biofilms, algal growth activities were not significantly impacted by surface hydrophobicity, which is consistent with previous studies where no/weak correlation were observed for long-time cultivation. Nevertheless, the formation and growth of mixotrophic microalgae biofilms were significantly correlated to surface hydrophobicity with higher hydrophobicity resulting in higher biomass accumulation. One underlying reason could be that mixotrophic microalgae biofilms are less vulnerable to light and nutrients limitations as autotrophic biofilms. The other explanation could be that, compared to autotrophic microalgae biofilms, mixotrophic microalgae biofilms have more stable and integrated biofilm structures (due to the extracellular polysaccharide accumulation/release triggered by the extra carbon flux), which can resist the shearing effect and carry the impact of initial colonization through longer growth period. This is a new discovery indicating that surface hydrophobicity should be taken into consideration when growing microalgae biofilms under mixotrophic conditions. For the impact of surface roughness, previous findings are not consistent. Studies have indicated that increasing surface roughness could either enhance biomass gains^22,72,73^ or had no significant impact^22,72^. Our results observed that surface roughness did not significantly affect the overall biomass yields for both autotrophic and mixotrophic microalgae biofilms. However, since only three degrees of roughness were used in this study, further research is needed to include more variety of surface roughness and surface pattern.

### Algal Feedstock Quality - Lipid and Ash Content

**Figure 3:**
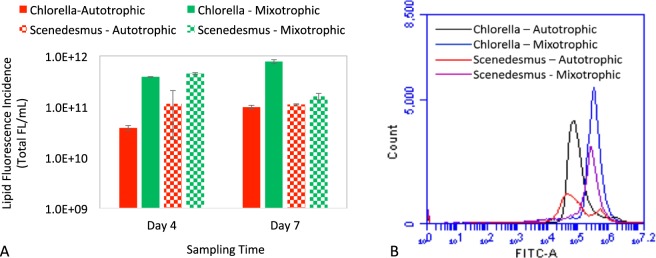
(**A**) Lipid productivities of microalgae biofilms under mixotrophic and autotrophic conditions in the MB3N medium. Mixotrophic microalgae biofilms exhibited much higher lipid productivity compared to autotrophic microalgae biofilms. The X-axis is the sampling time. The Y-axis is the total lipid fluorescence incidence in logarithmic scale. (**B**) Flow cytometry patterns of lipid fluorescence (FITC-A) in algal cells (4-day sample) stained with BODIPY 505/515 for neutral lipid productivity. Mixotrophic cultivation could promote lipid accumulation in algal cells compared to autotrophic cultivation. The X-axis (FITC-A) is the intensity of lipid fluorescence, with higher value indicating higher lipid concentration. The Y-axis is algal cell count with lipid fluorescence.

Lipid concentration and productivity were examined by using algal samples growing on the supporting material with medium hydrophobicity and roughness, i.e. PMMA with the treatment of 220-grit sandpaper, at two sampling times (4 and 7 days) when biofilms were in the stationary phase. The lipid productivity is expressed as total lipid fluorescence in one mL of algal sample (Total FL/mL). As shown in Fig. 3A, mixotrophic microalgae biofilms exhibited significantly higher (2- to 10-times higher of lipid fluorescence incidence, p < 0.001) lipid productivity, when compared to autotrophic microalgae biofilms. The enhancement of lipid accumulation was much more significant than that of biomass productivity. This was attributed to the increase of both biomass yield and the lipid concentration within algal cells. As shown in Fig. 3B, mixotrophic cultivation resulted in a shift of algal cells towards higher lipid content, which was evidenced by the lipid fluorescence intensity shifting towards the right of the FITC-A axis. It should be noted that, for *Scenedesmus dimorphus*, the lipid productivity under mixotrophic condition decreased in 7-day sample compared to 4-day sample. The reason could be that the lipid accumulation in *Scenedesmus dimorphus* occurred in the early stage of stationary phase (4 days).

Lipid concentration and productivity are critical for algal biofuel production. Studies of algal biofilms under autotrophic conditions have reported significantly lower lipid content within algal biomass than those cultivating algae planktonically^62^. One strategy to improve lipid accumulation is nutrition starvation. However, this approach is complicated and unpredictable because it is very challenging to balance the trade-off between biomass yield and lipid accumulation under these stress conditions^62^. The other way to increase lipid yield could be mixotrophic cultivation which is much easier to control and predict. Numerous studies of planktonic algae have demonstrated that mixotrophic cultivation could significantly increase lipid productivity. For instance, Liang *et al*. (2009) found maximum lipid productivity for *Chlorella vulgaris* under mixotrophic growth compared to autotrophic and heterotrophic conditions^44^. Likewise, Ryu *et al*. (2014) reported that *C*. *protothecoides* and *Ettlia sp*. yielded 28.7- and 17.3-fold higher lipid productivity in mixotrophic condition compared to photoautotrophic cultivation^45^. Our results, for the first time, demonstrate that mixotrophic cultivation can significantly enhance the lipid productivity of algal biofilms. This finding provides a new approach to addressing the challenge of low lipid productivity in algal biofilms.

Ash content analyses showed that mixotrophic growth could significantly reduce the ash content of algal biomass (p < 0.01). The average ash content of mixotrophic biofilms was about 45% lower compared to that of autotrophic biofilms (Table 1). The average ash content of *Chlorella vulgaris* was 9.1% (±0.5%) and 16.8% (±1.2%) for mixotrophic and autotrophic biofilms, respectively. The average ash content of *Scenedesmus dimorphus* was 8.5% (±0.5%) and 15.3% (±1.1%) for mixotrophic and autotrophic biofilms, respectively. Ash content is the other critical parameter that determines the quality of algal feedstock for various applications. Previous studies have indicated that one disadvantage of algal biofilms is their relatively high ash content (up to 30%) compared to planktonic algae^21^. Our study suggests that mixotrophic cultivation could reduce the ash content of algal biofilms. The underlying reason could be that, with the extra organic carbon supply, mixotrophic cultivation promotes lipid accumulation, thus reducing the overall ash content within the biomass. However, further research is warranted to test this cultivation strategy in outdoor environments.

**Table 1:** The average ash content of algal biomass in different cultivation conditions.

Medium	Algae	Ash content	
		Mixotrophic	Autotrophic
MB3N	*Chlorella*	9.1% (±0.5%)	16.8% (±1.2%)
	*Scenedesmus*	8.5% (±0.5%)	15.3% (±1.1%)
Primary Wastewater Effluent	*Chlorella*	6.9% (±0.6%)	13.5% (±1.9%)
	*Scenedesmus*	6.7% (±3.2%)	16.7% (±2.4%)

### Growth Activities of Microalgae Biofilm in Actual Wastewater

**Figure 4:**
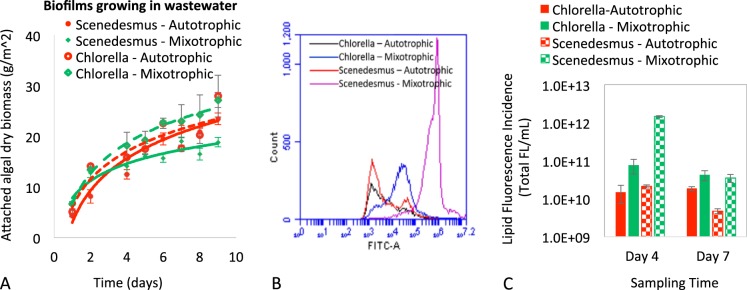
(**A**) Biomass productivities of microalgae biofilms in primary wastewater effluent. The biomass yields were not significantly different (P > 0.05) between autotrophic (without glucose) and mixotrophic (with the glucose) biofilms. (**B**) Flow cytometry patterns of lipid fluorescence (FITC-A) in algal cells (4-day sample). Mixotrophic cultivation could promote lipid accumulation in algal cells compared to autotrophic cultivation. The X axis (FITC-A) is the intensity of lipid fluorescence, with a higher value indicating higher lipid concentration. The Y axis is algal cell count with lipid fluorescence. (**C**) Lipid productivities of microalgae biofilms in primary wastewater effluent. Mixotrophic microalgae biofilms with glucose exhibited much higher lipid productivity compared to microalgae biofilms without glucose. The X-axis is the sampling time. The Y-axis is the total lipid fluorescence incidence in logarithmic scale.

Wastewater-algae integration is a sustainable and multi-functional approach for both bioenergy production and wastewater treatment in a single, integrated process^2,11,74–76^. To examine the applicability of growing mixotrophic microalgae biofilms in wastewater, we examined their growth activities in actual wastewater (primary effluent) by using the supporting material with medium hydrophobicity and roughness, i.e. PMMA with the treatment of 220-grit sandpaper. As shown in Fig. 4A, biomass yields between autotrophic and mixotrophic biofilms were not significantly different (P > 0.05). The underlying reason could be that organic carbons naturally presenting in actual wastewater can support mixotrophic growth, which attenuates the impact of the addition of extra organic carbon (glucose). The average biomass productivity was 3.9 and 3.3 g/m^2^-day for *Chlorella* and *Scenedesmus* respectively. This productivity is very competitive to commonly reported yields of microalgae biofilms in wastewater (0.5–3 g/m^2^-day)^54^.

However, the addition of glucose could significantly enhance (p < 0.001) lipid accumulation for both algae species (Fig. 4B,C). Algal cells growing in wastewater with glucose had much higher lipid concentrations, which was evidenced by stronger lipid fluorescence intensity in the FITC-A axis (Fig. 4B). This resulted in significantly higher overall lipid productivity when growing algae with the addition of glucose (Fig. 4C), even though biomass yields were similar in the two cultivation conditions. Particularly for *Scenedesmus dimorphus*, the lipid yield in mixotrophic condition was 68-time (4-day sample) and 10-time (7-day sample) higher than that in autotrophic cultivation. Similar to algae growing in MB3N medium, the lipid productivity of *Scenedesmus dimorphus* decreased in the 7-day sample compared to the 4-day sample. In addition, the ash content of algal biomass cultivated in wastewater with glucose was 60% lower than that of algal biomass growing in wastewater without glucose (Table 1). The average ash contents of *Chlorella vulgaris* were 6.9% ± 0.6% (with glucose), and 13.5% ± 1.9% (without glucose); and the average ash contents of *Scenedesmus dimorphus* were 6.7% ± 3.2% (with glucose) and 16.7% ± 2.4% (without glucose).

Wastewater, currently underused, could be one of the most favourable resources for algae feedstock production, because it (1) provides ample supply of nutrients and water; (2) can support a large capacity for biofuel production, 5 billion gallons of algal biofuel per year could be generated with municipal wastewater in the U.S.; (3) reduces costs for both wastewater treatment and algal biofuel production, up to 70% of activated sludge treatment costs and 30–50% of the algal biofuel costs; and (4) can be integrated into existing public infrastructure, rather than creating new isolated industrial system^2,11,74–77^. However, wastewater-algae integration has not yet been achieved at large-scale due to a number of challenges, particularly the poor biomass quality (low lipid content and high ash content)^2,5^. Studies have found that wastewater-based mixotrophic cultivation of planktonic algae, by adding extra organic carbons with low molecular weight (such as glucose, glycerol, acetate, and volatile fatty acids), can significantly enhance algal biomass productivity and quality^45,49,78,79^. Our results suggest that promoting mixotrophic growth via the addition of organic carbons could be a feasible solution for cultivating microalgae biofilms in wastewater because it can facilitate the metabolism of lipid synthesis in algal cells, resulting in higher lipid productivity and lower ash content. This approach is compliant with established wastewater treatment practices wherein extra organic carbons are usually added to enhance biological treatment. In addition, many low-molecular organic carbons such as glycerol, acetate, and volatile fatty acids are byproducts from other industries which are easy to be obtained at low-cost^45,49,78,79^.

In summary, this study characterized the formation and growth activities of microalgae biofilm under different conditions. The results prove an innovative cultivation strategy, mixotrophic microalgae biofilm, to cultivate algae which leads to improved productivity and quality (higher lipid accumulation and lower ash content) of algal biofuel feedstock. Different from other algae cultivation methods such as open ponds, closed photo-bioreactors, and autotrophic biofilms, mixotrophic microalgae biofilm is capable of harnessing the benefits of both mixotrophic growth’s high efficiency and biofilm’s low harvesting cost to achieve high productivity/quality at low cost. In addition, our results demonstrate the applicability of integrating this novel cultivation strategy with wastewater. This work opens a new possibility to solve the long-lasting challenges of algal biofuel production, i.e., low productivity and high cost of feedstock cultivation. However, due to the very limited studies on mixotrophic microalgae biofilms, further research is warranted to better understand the mechanisms underlying the enhanced biofilm formation and biomass/lipid accumulation of mixotrophic microalgae biofilm, particularly those mechanisms at genetic levels, for designing such cultivation systems with maximum efficiency. In addition, other low-cost organic carbon sources such as glycerol, acetate, and volatile fatty acids should be included to investigate their impact on the growth activity of mixotrophic microalgae biofilm for cost-efficient cultivation. These three low-cost carbon sources are byproducts from other industries and have been used for mixotrophic algae cultivation^45,49,78,79^.

## Materials and Methods

### Algae Species, Biofilm Supporting Material, and Growth Medium

*Chlorella vulgaris* and *Scenedesmus dimorphus* (UTEX culture collection of algae at the University of Texas at Austin) were chosen as the model algae in this study due to their rich oil content, and their capability of forming biofilms and growing under mixotrophic conditions^21,75,80–83^.

Four different biofilm supporting materials, stainless steel (SS), polypropylene (PP), acrylic (PMMA), and polycarbonate (PC) with treatment of different grit of sandpaper were used as supporting substrates to examine the impacts of cell-surface properties on the formation and growth of algal biofilms. For PP, PMMA and PC, materials were treated with 60-, 220- and 400-grit sandpaper respectively to represent different roughness. For SS, only 220-grit sandpaper was used for the treatment due to the difficulty of sandpaper treatment. SS treated with 220-grit sandpaper was considered as rough; while SS without sandpaper treatment was considered as smooth. The hydrophobicity of the substrate surface was determined by the KSV contact angle measurement system. Two types of growth medium were used: Modified Bold 3 N Medium (MB3N) and actual wastewater (primary effluent) collected from the Detroit’s Wastewater Treatment. For mixotrophic cultivation, 1 g/L of glucose was added to the growth medium. Before algae cultivation, wastewater was filtered through a filter cloth or coffee paper to remove large particles. Please see Support Information (SI) for the detailed composition of culture medium.

### Experimental Setup

For each algae species and growth medium, four sets of aquariums (two replicates for autotrophic condition and the other two replicates for mixotrophic condition) containing 1.5 × 1.5 inch coupons were used to examine the formation and growth activities of microalgae biofilm under different conditions (Fig. S2). Briefly, 1.5 × 1.5 inch coupons from different materials with different sandpaper treatments were stabilized in the bottom of the aquarium by an initial inoculation of 1 L algal culture with the same Chlorophyll-a concentration (OD680 value of 0.1 ± 0.01). Chlorophyll a concentration was measured with the optical density at 680 nm wavelength (OD680) by using the spectrophotometer (Thermo Scientific). Two growth conditions (autotrophic and mixotrophic) were included, with the addition of 1 g/L of glucose for mixotrophic cultivation. Growth conditions were maintained to maximally simulate the natural environment: room temperature (25 °C), no air or CO_2_ bubbling, 12 hrs/12 hrs of light/dark cycle with a light intensity of 100 µmol.m^−2^ s^−1^. The dimension of the aquariums is W = 25.4 cm, L = 50.8 cm and the depth of the fluid in the aquarium was 4 cm. The total volume of fluid in each set of aquariums was 10 litters. The aquariums was operated as a continuous flow model with a flow rate of 30 mL/s.

### Algal Biofuel Feedstock Characterization

At each sampling time (1–9 days), two coupons for every material and treatment from each of the two setups were taken out. Algae attached to the measuring area (one square inch) were analyzed for dry biomass productivity, ash content and lipid content. For autotrophic biofilms, the sampling time was extended to 13 or 15 days due to the longer lag phase and slower growth rate. Algae attached to the measuring area (one square inch) in the coupon was washed with 50 ml of distilled water.

BD Accuri™ C6 Flow Cytometer was used for algal cell count and lipid analysis according to the method established by Hallenbeck *et al*.^84^. Specifically, algal cell count was measured by detecting the fluorescence of chlorophyll *a* with the result of cells/mL. For lipid analysis, lipid-binding dye, BODIPY 505/515, was used. 10 µL of 1.25 µM BODIPY dye was added to 990 µL of algal sample. The mixture was mixed well before the analysis. The 515 filter in channel 1 (FL1) was used for the detection of lipid-binding dye signal. The overall lipid productivity was determined by the sum of fluorescent intensity in all algal cells with lipid fluorescence reading. The results were expressed as total fluorescent units per mL sample (Total FL/mL). This method has been used by many other studies and its results correlates very well with the gravimetric determination of algal lipids (R^2^ = 0.8–0.99), which is also confirmed by our results (R^2^ = 0.96, Fig. S3). Moreover, this method can not only generate information of total lipid productivity but also allow the examination of lipid concentration on a cellular level of changes^84^. Algal dry biomass and ash content were measured as the weight fraction after drying and the residual fraction after combustion, respectively^85^. Algae were collected using filters (1.2 µm of pore size) and then dried using a hot-air oven at 105 °C until no weight change was observed. Ash content was then determined by furnace burning at 600 °C.

### Statistical analysis

IBM SPSS software (IBM Corp., Armonk, New York) was used for statistical analyses. ANOVA (analysis of variance) was used to analyze differences of algal dry biomass, lipid productivity and ash content respectively among different cultivation condition including growth mode, algal species, growth medium, and cell-surface properties. Pearson’s correlation coefficient was calculated for the correlation between algal dry biomass and surface hydrophobicity and roughness respectively. For all statistical analyses, p-values < 0.05 are considered to be significant.

## Electronic supplementary material

Supporting Information
