# Supplementary material for: Mixotrophic Microalgae Biofilm: A Novel Algae Cultivation Strategy for Improved Productivity and Cost-efficiency of Biofuel Feedstock Production
Source: Sci Rep. 2018 Aug 21;8:12528. doi: 10.1038/s41598-018-31016-1 (PMC6104096; doi:10.1038/s41598-018-31016-1)
Supplement: Supplementary file 1 — Supporting Information [file 41598_2018_31016_MOESM1_ESM.docx]

**Mixotrophic Microalgae Biofilm: A Novel Algae Cultivation Strategy for Improved Productivity and Cost-efficiency of Biofuel Feedstock Production**

Supporting Information

Javad Roostai ^a^, Yongli Zhang ^a^[[1]](#footnote-1)^*^, Kishore Gopalakrishnan ^a^, Alexander Ochocki ^b^

^1^ Civil and Environmental Engineering, Wayne State University, 5050 Anthony Wayne Dr., Detroit, MI 48202.

^2^ Biological Sciences, Wayne State University, 5047 Gullen Mall, Detroit, MI 48202.

**1. Figure S1: Hydrophobicity measurement of substrate materials**


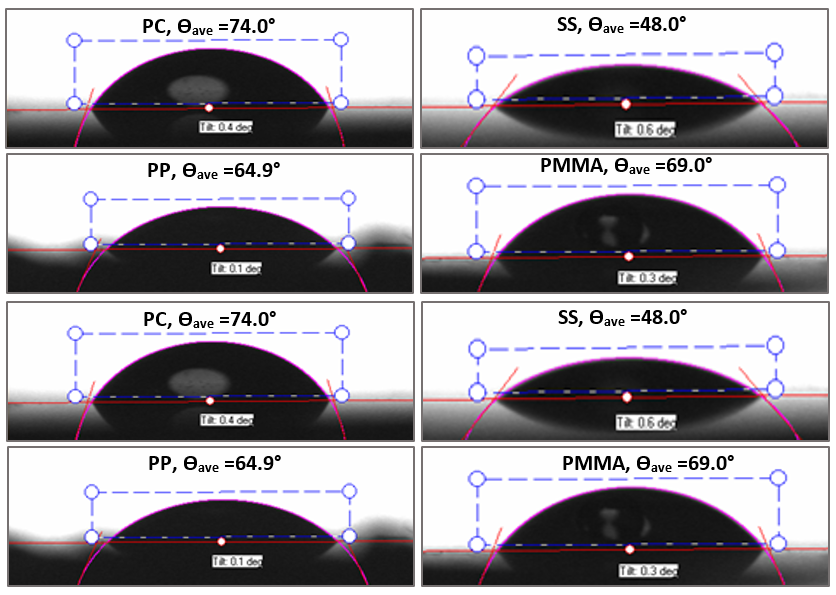


**2. Growth Mediums**

**2.1. MB3N Medium**

General-purpose medium used for axenic cultures, especially blue-green and red algae.

**2.1.1 Ingredients of Modified Bold 3N Medium**

|  |  | **#** | **Component** | **Amount** | **Stock Solution  Concentration** | **Final Concentration** |
| --- | --- | --- | --- | --- | --- | --- |
|  |  | 1 | NaNO_3_ | 30 mL/L | 10 g/400mL dH2O | 8.82 mM |
|  |  | 2 | CaCl_2_**·**2H_2_O | 10 mL/L | 1 g/400mL dH2O | 0.17 mM |
|  |  | 3 | MgSO_4_**·**7H_2_O | 10 mL/L | 3 g/400mL dH2O | 0.3 mM |
|  |  | 4 | K_2_HPO_4_ | 10 mL/L | 3 g/400mL dH2O | 0.43 mM |
|  |  | 5 | KH_2_PO_4_ | 10 mL/L | 7 g/400mL dH2O | 1.29 mM |
|  |  | 6 | NaCl | 10 mL/L | 1 g/400mL dH2O | 0.43 mM |
|  |  | 7 | [P-IV Metal Solution](http://web.biosci.utexas.edu/utex/mediaDetail.aspx?mediaID=127) | 6 mL/L |  |  |
|  |  | 8 | [Soilwater: GR+ Medium](http://web.biosci.utexas.edu/utex/mediaDetail.aspx?mediaID=47) | 40 mL/L |  |  |
|  |  | 9 | [Vitamin B_12_](http://web.biosci.utexas.edu/utex/mediaDetail.aspx?mediaID=123) | 1 mL/L |  |  |
|  |  | 10 | [Biotin Vitamin Solution](http://web.biosci.utexas.edu/utex/mediaDetail.aspx?mediaID=131) | 1 mL/L |  |  |
|  |  | 11 | [Thiamine Vitamin Solution](http://web.biosci.utexas.edu/utex/mediaDetail.aspx?mediaID=130) | 1 mL/L |  |  |

**2.1.2 Ingredients of metal solution**

|  | \| **#** \| **Component** \| **Amount** \| **Final Concentration** \| \| --- \| --- \| --- \| --- \| \| 1 \| Na_2_EDTA·2H_2_O \| 0.75 g/L \| 2 mM \| \| 2 \| FeCl_3_**·**6H_2_O \| 0.097 g/L \| 0.36 mM \| \| 3 \| MnCl_2_**·**4H_2_O \| 0.041 g/L \| 0.21 mM \| \| 4 \| ZnCl_2_ \| 0.005 g/L \| 0.037 mM \| \| 5 \| CoCl_2_**·**6H_2_O \| 0.002 g/L \| 0.0084 mM \| \| 6 \| Na_2_MoO_4_**·**2H_2_O \| 0.004 g/L \| 0.017 mM \| |
| --- | --- | --- | --- | --- | --- | --- | --- | --- | --- | --- | --- | --- | --- | --- | --- | --- | --- | --- | --- | --- | --- | --- | --- | --- | --- | --- | --- | --- | --- |

**2.1.3 Soil Water: GR+ Medium**

The basic garden-type soilwater; includes a pinch of CaCO3, which is added to the soil and water prior to steaming; suitable for most phototrophic freshwater algae.

1). Combine all components listed.

2). Cover the medium container and steam for 2 consecutive days, 3 hours on each day. Pasteurization is a gradual rising of temperature to approximately 95°C in 15 minutes. Then increased just over 98°C for the 3 hour duration. Cooling occurs gradually at room temperature.

3). Refrigerate 24 hours or more and bring to room temperature before using.

Ingredients of soil water

| **Component** | **Amount** | **Final Concentration** |
| --- | --- | --- |
| Green house soil | 1 tsp/200 mL water |  |
| CaCO3 (optional) | 1 mg/200 mL dH2O | 0.05 mM |

**2.2. Primary wastewater effluent**

Primary wastewater effluent was obtained from the Detroit Water and Sewerage Department. The general C/N/P composition of primary wastewater effluent is BOD (155-286 mg/L), TN (26 – 75 mg/L) and TP (6 – 12 mg/L).

**3. Experimental Setup**

**A**
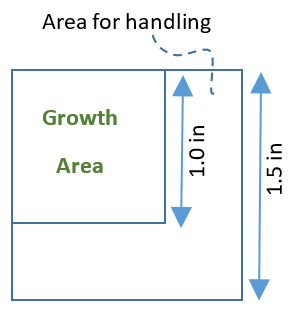
 **B**
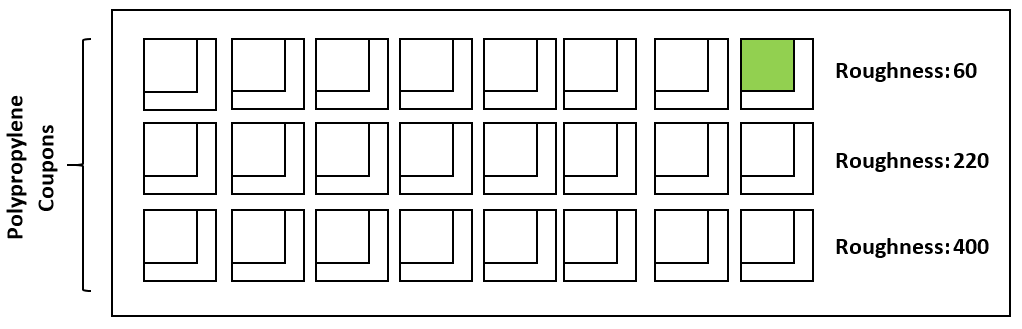


**C**
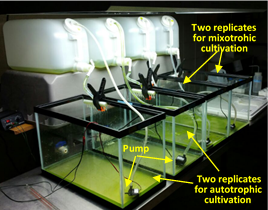
 **D**
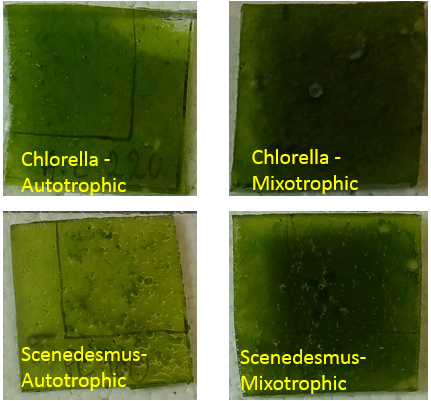


**Figure S2:** Experimental design and setup. **A**, coupons for biofilm growth; **B**, the aquarium that contains coupons from different material with different roughness treatment; **C**, experimental set up; **D**, biofilm samples growing on coupons (4-day samples in MB3N medium).

System Dimension and Flow Rate:

h= 4 cm

w= 25.4 cm

w= 50.8 cm

Q= 1000 ml in 29 to 39 second = Average 34 second

Average Q = 1000 ml/34 second = 2.94*10^-5^m^3^/s = 30 mL/s

**4. Lipids analyses and calibration**

For lipids analyses, we have performed an experiment to compare and calibrate the methods of gravimetric analysis of lipids and flow cytometer analysis. The gravimetric analysis of lipids was performed according to the methodology described by Carpio et al., 2014 ^1^. Our results indicate a good correlation between the results from these two different methods (Figure S3), which is consistent with other studies ^2^.

**Figure S3:** The correlation between lipid measurements obtained from flow cytometer analysis (BODIPY fluorescence incidence) and gravimetrical determination.

**6. References**

1. Carpio, R. B., De Leon, R. L., and Martinez-Goss, M. R. Growth, lipid content, and lipid profile of the green alga, Chlorella Vulgaris Beij., under different concentrations of Fe and CO_2_. *Journal of Engineering Science and Technology* **January,** 19-30 (2015).

# 2. Rumin, J., Bonnefond, H., Saint-Jean, B., Rouxel, C., Sciandra, A., Bernard, O., Cadoret, J. P., Bougaran, G. The use of fluorescent Nile red and BODIPY for lipid measurement in microalgae. *Biotechnology for Biofuels* 8: 42 (2015).

1. * Corresponding author: [zhangyl@wayne.edu](mailto:zhangyl@wayne.edu) [↑](#footnote-ref-1)
